# Improving C1-C2 Complex Fusion Rates: An Alternate Approach

**DOI:** 10.7759/cureus.1887

**Published:** 2017-11-29

**Authors:** Samer S Ghostine, Paul E Kaloostian, Christ Ordookhanian, Sean Kaloostian, Parham Zarrini, Terrence Kim, Stephen Scibelli, Scott J Clark-Schoeb, Srinath Samudrala, Carl Lauryssen, Amandip S Gill, Patrick J Johnson

**Affiliations:** 1 Neurological Surgery, University of California, Riverside School of Medicine; 2 Neurological Surgery, University of California, Irvine School of Medicine; 3 Cardiology, University of Miami, Medical School; 4 Orthopedic Surgery, Cedars-Sinai Medical Center; 5 Neurological Surgery, Memorial Neurospine; 6 Spine Surgery, New Jersey Spine Center; 7 Neurological Surgery, Pomona Valley Medical Center; 8 Neurological Surgery, St. David’s Round Rock Medical Center; 9 Institute for Spinal Disorders, Cedars-Sinai Medical Center

**Keywords:** c1-c2, atlantoaxial, atlantoaxial fusion, aai, spinal instability, spinal instability, intraarticular, fusion, nerve root, atlas axis

## Abstract

The surgical repair of atlantoaxial instabilities (AAI) presents complex and unique challenges, originating from abnormalities and/or trauma within the junction regions of the C1-C2 atlas-axis, to surgeons. When this region is destabilized, surgical fusion becomes of key importance in order to prevent spinal cord injury. Several techniques can be utilized to provide for the adequate fusion of the atlantoaxial construct. Nevertheless, many individuals have less than ideal rates of fusion, below 35%-40%, which also involves the C2 nerve root being sacrificed. This suboptimal and unavoidable iatrogenic complication results in the elevated probability of complications typically composed of vertebral artery injury. This review is a retrospective analysis of 87 patients from Cedars Sinai Medical Center in Los Angeles, California, who had the C1-C2 surgical fusion procedure performed within the time frame from 2001 to 2008, with a mean follow-up period of three years. These patients had presented with typical AAI symptoms of fatigability, limited mobility, and clumsiness. Diagnosis of C1-C2 instability was documented via radiographic studies, typically utilizing computed tomography (CT) scans or x-rays. All patients had bilateral C1 lateral masses and C2 pedicle screws. In addition, the C1-C2 joint was accessed by retracting the C2 nerve root superiorly and exposing the joint by utilizing a high-speed burr. The cavity that is developed within the joint is packed with local autologous bone from the cephalad resection of the C2 laminae. Fusion of the C1-C2 joint was achieved in all patients and a final follow-up was conducted approximately three years postoperative. Of the 87 patients, two presented with occipital headaches resulting from the C1 screws impinging on the C2 nerve root. The issue was rectified by removing instrumentation in both patients after documenting complete fusion via radiographic studies, with complete resolution of symptoms. No vertebral artery or spinal cord injuries were reported as a result of the minor complication. Overall, we aim to describe a safe and reliable alternative technique to fuse C1-C2 instability by focusing on intra-articular arthrodesis complementing instrumentation fixation. This methodology is advantageous from a biomechanical standpoint secondary to axial loading, as well as the large surface area available for arthrodesis. Additionally, this technique does not involve the resection of the C2 nerve root, resulting in low risk for vertebral artery or spinal cord injury.

## Introduction and background

Atlantoaxial instability (AAI) is a complex condition that must utilize surgical treatment to provide rigid fixation with a high rate of fusion (>95%). Through the advancement of medical technology and the influx of new medical research, the surgeon’s methodology of treatment is ever evolving, especially over the past few decades. Early on, stabilization of the C1-C2 complex (CCC) was achieved with instrumentation involving cable-graft wires that yielded suboptimal fusion rates without postoperative halo support [1-5]. Later on, Magerl and Seeman described the challenging C1-C2 transarticular screw stabilization, which yielded a satisfactory fusion rate without the need for a post-operative halo; however, a significant increase in the potential rate of surgical complication did arise, which included the risk of vertebral artery injury [6-20]. Subsequently, there are additional techniques that have been described in the literature, which involve a C1 transarticular screw combined with a C2 pedicle, pars interarticularis, or translaminar screws that can achieve an acceptable fusion rate without jeopardizing the rate of surgical success and minimizing postoperative complications [5,21-24]. Recently, a variant of the Harms and Goel technique was published in which C2 nerve roots were sacrificed bilaterally, allowing for the surgical insertion of an allograft spacer within the C1-C2 joint, while distracting a previously placed bilateral C1 lateral mass and C2 pedicle or pars interarticularis screws [25]. While this technique preferentially allows for a high rate of CCC fusion, there is a slight chance (<34%) that postoperative occipital headaches can develop, which are associated with the C2 nerve root sacrifice. In our paper, we present a technique where we perform complete arthrodesis along the entire C1-C2 joint, complementing instrumentation fixation, without sacrificing the C2 nerve roots. Within the patients we treated, we report a 100% rate of fusion, but we do forewarn that our rate of fusion may deviate within larger patient sample sizes due to unmanipulable iatrogenic complicative variables and unforeseen surgical complications, which are present within all surgical practices.

## Review

Methods

Case-Study Description

Within this retrospective study of 87 patients, all patients had some degree of C1-C2 complex instability. This retrospective study was conducted within one large academic center from 2001 to 2008 with Institutional Review Board approval. Each of these patients, to some degree (as mentioned earlier), had a form of C1-C2 instability, necessitating the need for C1-C2 stabilization and fusion. Indications frequently included, but were not restricted to os odontoideum (OO) and odontoid fractures, both acute and nonunion, C1-C2 instability, C2 mass formation, and traumatic C1-C2 fracture. These indications were evaluated through radiographic studies utilizing the professional expertise of a radiologist to review the various computed tomography (CT), x-ray, and magnetic resonance imaging (MRI) scans both preoperatively and postoperatively [22-27]. 

Surgical Procedure Methodology

Patients were positioned in the prone position and placed into the Mayfield head stabilization apparatus. The exposure of the C1 and C2 segments was accessed posteriorly and the C2 roots were bilaterally exposed and altered from anatomically normal positioning to reveal the C1-C2 joint without severing the nerve root. Partial laminectomy at the C2 joint was then completed and the spinous segment of the C2 was where the drilling occurred for the placement of a pedicle screw. The entirety of the complex was locked together by a rod system and screws. Utilizing a torque driver, all screws and rods were firmly locked into place together.

Results

Post-operative outcomes for all patients were verified through radiographical studies over a mean of three years and noted a 100% rate of fusion, except for two patients who experienced postoperative complications and underwent additional surgical procedures [28]. Of the two reports of postoperative headaches, surgeons noted the cause was likely due to screw impingement on the C2 nerve root. In each of these cases, the screws were removed safely, with complete resolution of patient pain. In all cases, no spinal cord injury or vertebral artery injury was noted, along with no clinically significant manifestations of neurological deficits with any of these patients postoperatively, as determined by review of the clinical documentation [29]. The radiological studies conducted allowed for the interpretation of postoperative scans for the assessment of fusion and the integrity of instrumentation. Each patient was evaluated by a review of the clinical documentation within specific patient charts, and it was deduced that such a surgical procedure was indeed optimal and recommended among neurological surgeons, aided by radiological studies [30-35].

Discussion

Evolution of the Atlantoaxial Fusion

Preliminary descriptions of posterior atlantoaxial fusion were conducted by Gallie, which involved the placement of a notched bone graft between the posterior arch of C1 and the medial lamina and the spinous process of C2 [36]. This surgical construct was functionally limited due to its diminished ability to resist rotational stress, resulting in the need for a rigid postoperative orthosis. To provide enhanced rotational stability, Brooks performed bilateral, interlaminar bone grafts [1]. Within the two surgical procedures discussed, performed by Gallie and Brooks, bone grafts were secured by sublaminar wires between C1-C2. However, the placement of such sublaminar wires provided for an increased chance of injury to neural structures within the spinal canal. These suboptimal results and surgical complications prompted the development of novel techniques to replace the sublaminar wiring at C2 with a loop of wire around the C2 spinous region [2,37]. The Locksley intersegmental tie-bar technique added a posterior stabilization plate involving an additional wire joined to the posterior C1 and C2 spinous processes [36]. Along with the bilateral onlay grafts, this plate gave three-point fixation and greatly increased stabilization [38]. Interlaminar hooks, called Halifax clamps or Knot’s rods, were developed by Tucker [39]. These interlaminar devices were used to secure bilateral interlaminar bone grafts similar to Brooks' fusion and did not require the passing of sublaminar wires. However, the dislodgment of the clamps was a frequently observed surgical complication, most likely attributed to the round shape of the posterior arch of C1. A halo orthosis was suggested to help prevent clamp dislodgment. When Magerl described the posterior transarticular screw, this was a major breakthrough for the field of neurosurgery, as it meant immediate and adequate stabilization of the fusion, replacing and rendering useless the need for postoperative external orthosis [36]. Like Barbour’s anterior transarticular screw, this technique did not require intact posterior arches at C1 and C2, except in cases where a Gallie-type bone graft and wiring was added to provide three-point fixation. Olerud et al. described an alternative method for providing three-point fixation by passing the transarticular screw through a plate that was attached to C1 by a claw [38]. More recently, Matsumoto supplemented the transarticular screws with autograft-containing titanium mesh cages, which were wired bilaterally in a manner similar to the Brooks method [39]. Today, bilateral polyaxial screws and rods are commonly used for atlantoaxial fusion, popularized by Harms and Melcher [16]. The screws are inserted into the lateral mass at C1 and the pedicles or pars at C2. Although structural grafting is not necessary, an onlay graft may be added to assist the fusion process. This technique has permitted C1-C2 instrumentation in patients with fixed C1-C2 subluxation and with an abnormal vertebral artery anatomy. However, C2 pedicle screws may violate the foramen transversarium, in which crossed translaminar C2 screws have been suggested as an alternative route [19].

The C1-C2 Joint

The C1-C2 articular joint distinguishes itself from the family of spinal joints by its integrating roles of intervertebral discs and facet joints of other spinal levels. It is horizontal and carries most of the head weight like all spinal intervertebral discs do. It is ventral to the spinal nerves and is able to assume a flexion-extension motion of 12 degrees like other cervical facet joints. However, unlike other spinal facet joints, it is capable of allowing for 47 degrees of rotational motion or 50% of the cervical spinal rotational motion capacity [20-21]. Being a small joint with a significant contribution to motion and agility, it is likely due to degenerative changes onset by the inflammatory pannus rheumatoid arthritis (RA), which ultimately lead to its instability. The instability of the joint is often clinically symptomatic, and a surgical fusion is warranted and, in most cases, necessary for the lividity of the patient [40-43].

C1-C2 Arthrodesis

**Figure 1:**
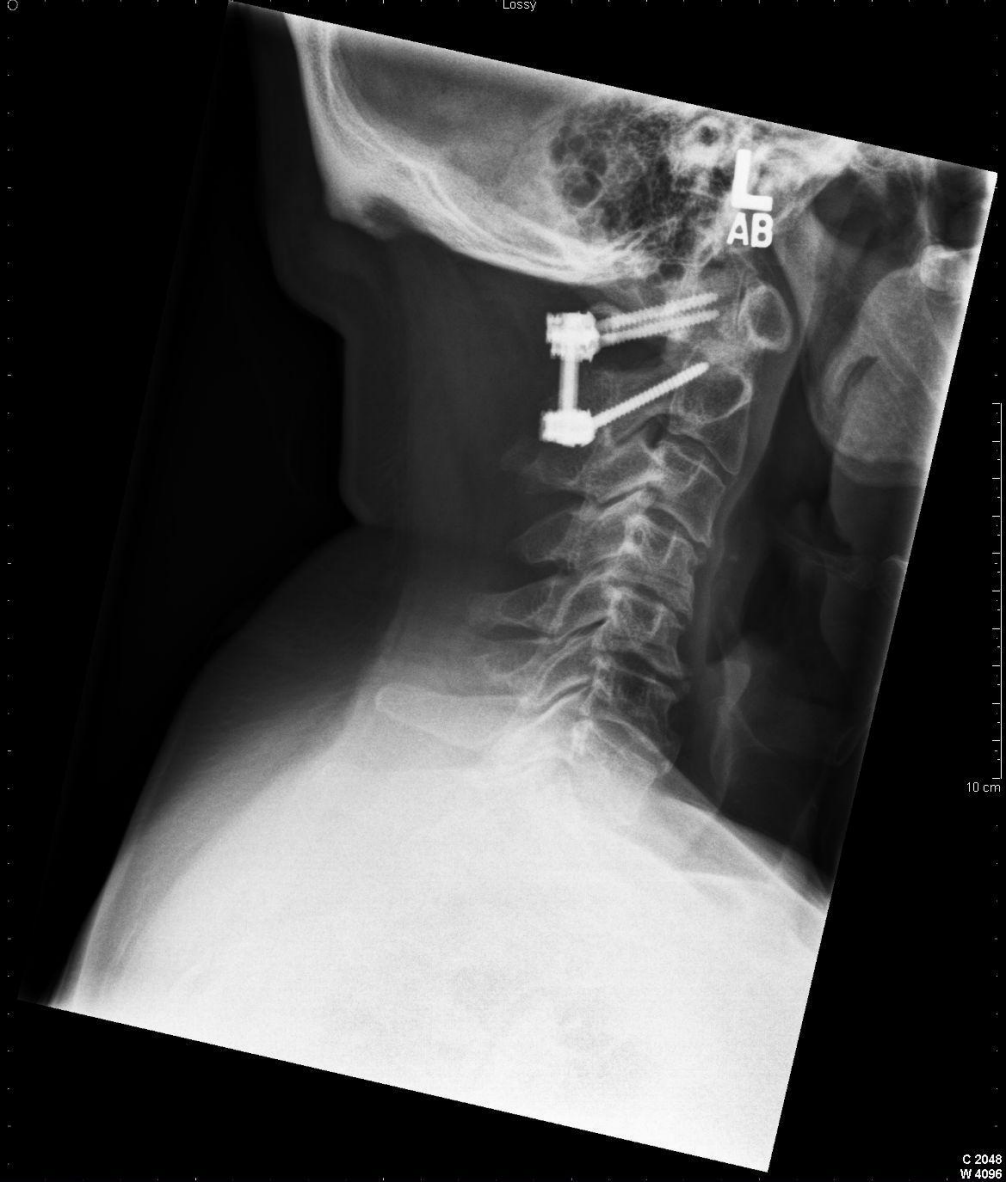
Example C1-C2 instrumentation is noted with appropriate placement of C1 lateral mass screws and C2 pedicle screws

**Figure 2:**
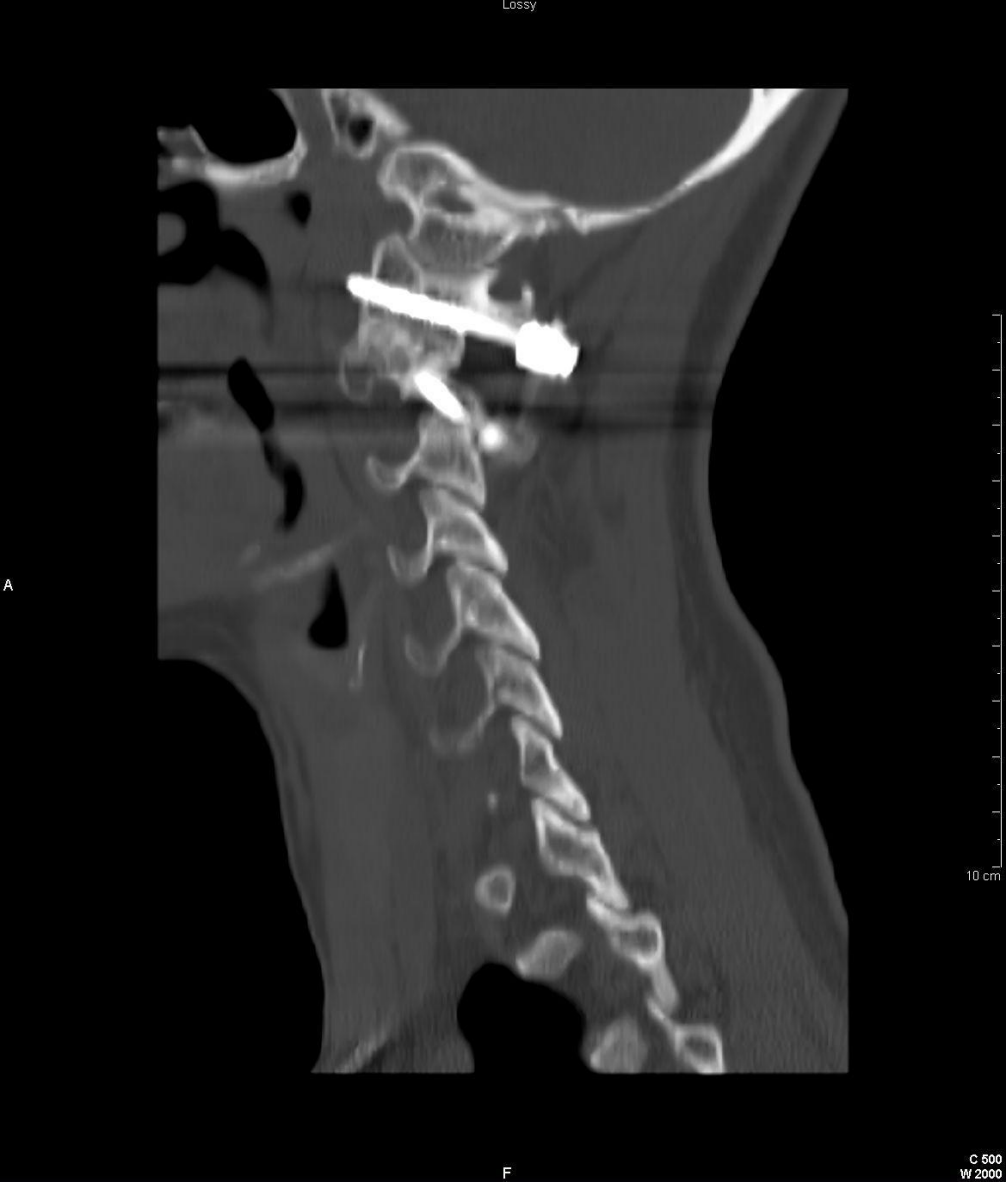
C1-C2 instrumentation with notable fusion, as seen by the bridging bony fusion mass post the arthrodesis of C1-C2 joints

**Figure 3:**
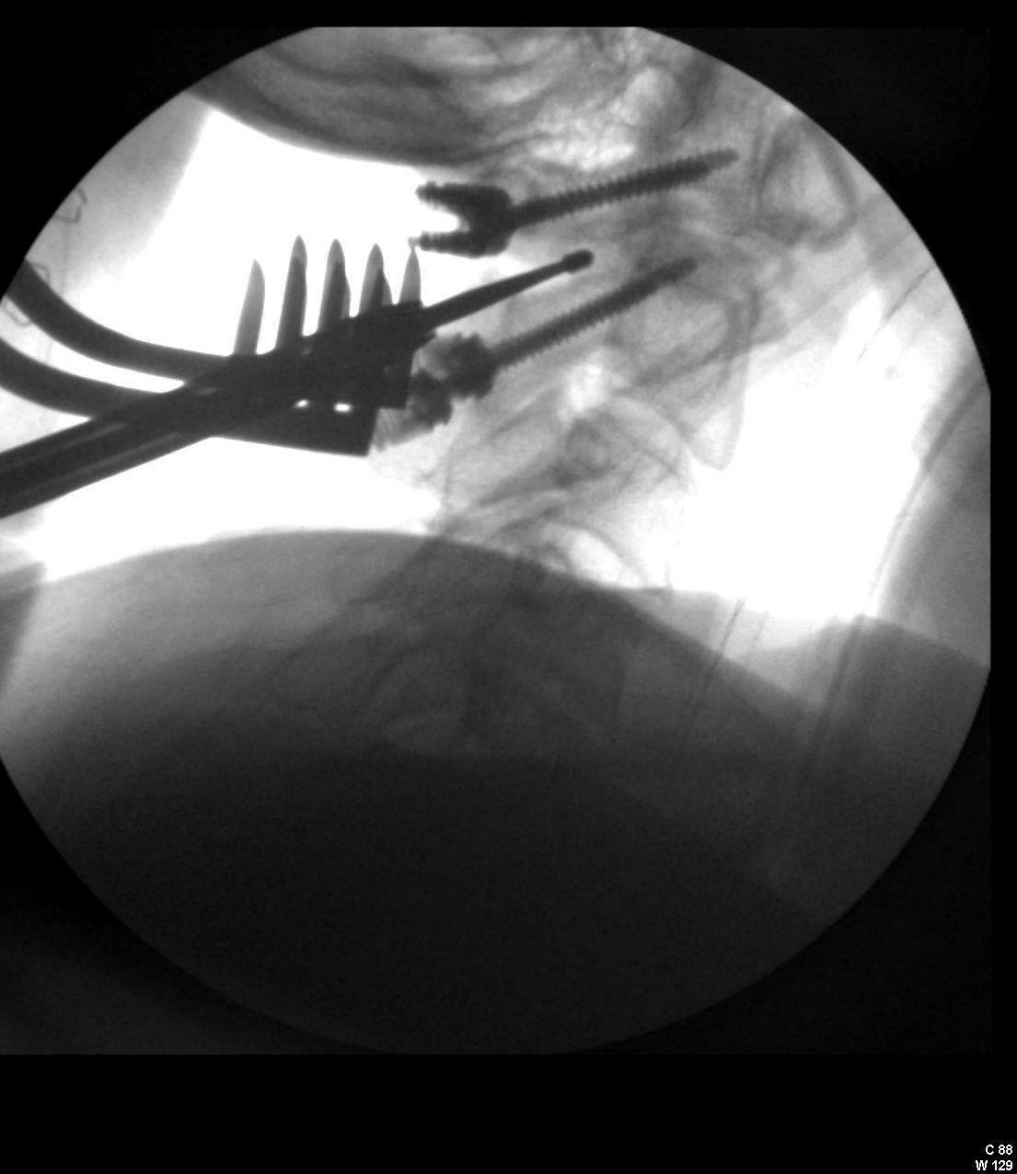
Intraoperative image post instrumentation as arthrodesis using high-speed drill is being performed along C1 and C2 as well as the joints between C1 and C2 bilaterally for fusion

Surgical fusion of mobile, unstable, or painful joints has been widely performed in the past and continues to be done; however, through the advancement of surgical technologies, former highly invasive techniques can be substituted by less invasive and overall better procedures. The fusion of the C1-C2 joint is routinely performed as a treatment of instability. Typically, in order to fuse the C1-C2 joint, spinal surgeons performed either an onlay fusion in the C1-C2 interlaminar space with or without the use of graft material or placed a graft in the interarticular C1-C2 joint in addition to instrumentation to immobilize the joint [1-2,20,44]. We describe complete arthrodesis at the CCC by the use of curettes and a high-speed drill. The curettes are used to identify the C1-C2 joints and allow for the removal of all cartilage along the joints [45]. A high-speed drill is used to then complete the arthrodesis at the CCC [46]. The subsequent removal of cartilage and roughing up of the CCC bilaterally allows for an increased fusion rate and acts as a scaffold for the placement of allograft and/or autograft material (Figures 1-3) in addition to the instrumentation at C1 and C2 bilaterally [47-50].

## Conclusions

Within our review, we describe an alternate surgical methodology that tremendously enhances the success rates of C1-C2 fusion. Our proposed methodology ensures a safe and reliable alternative technique to fuse the C1-C2 Complex (CCC) by performing an intraarticular arthrodesis. This is advantageous from a biomechanical standpoint secondary to axial loading and the large surface area available for arthrodesis. Additionally, this technique does not involve the resection of the C2 nerve root and results in low risks for vertebral artery or spinal cord injury. Overall, this is a method that we believe should be implemented in the skillset of every neurosurgeon when attempting a C1-C2 fusion. The tremendous outcomes and patient satisfaction far surpass the success rates of older methodologies.
